# Amplified Plasmonic Forces from DNA Origami-Scaffolded Single Dyes in Nanogaps

**DOI:** 10.1021/acs.nanolett.3c01016

**Published:** 2023-06-26

**Authors:** Sara Rocchetti, Alexander Ohmann, Rohit Chikkaraddy, Gyeongwon Kang, Ulrich F. Keyser, Jeremy J. Baumberg

**Affiliations:** † Nanophotonics Centre, Department of Physics, Cavendish Laboratory, University of Cambridge, Cambridge CB3 0HE, England, U.K.; ‡ School of Physics and Astronomy, University of Birmingham, Edgbaston, Birmingham B15 2TT, England, U.K.

**Keywords:** plasmonic nanocavity, DNA origami, quantum emitters, metal nanoparticle, picocavities, surface-enhanced Raman spectroscopy

## Abstract

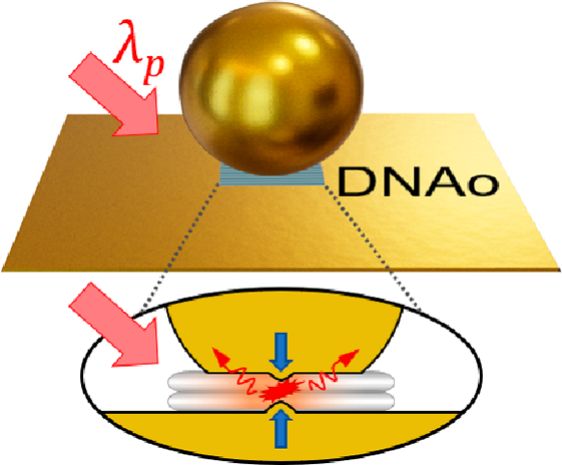

Developing highly enhanced plasmonic nanocavities allows direct observation of light–matter interactions at the nanoscale. With DNA origami, the ability to precisely nanoposition single-quantum emitters in ultranarrow plasmonic gaps enables detailed study of their modified light emission. By developing protocols for creating nanoparticle-on-mirror constructs in which DNA nanostructures act as reliable and customizable spacers for nanoparticle binding, we reveal that the simple picture of Purcell-enhanced molecular dye emission is misleading. Instead, we show that the enhanced dipolar dye polarizability greatly amplifies optical forces acting on the facet Au atoms, leading to their rapid destabilization. Using different dyes, we find that emission spectra are dominated by inelastic (Raman) scattering from molecules and metals, instead of fluorescence, with molecular bleaching also not evident despite the large structural rearrangements. This implies that the competition between recombination pathways demands a rethink of routes to quantum optics using plasmonics.

The interplay of energy between a light emitter and its environment modifies its spontaneous emission rate through the Purcell effect. Placing a quantum emitter in a photonically structured environment such as an optical cavity or photonic crystal can enhance the light–matter interaction enough to enter the strong-coupling regime.^1−5^ By using metallic nanostructures, light can be even more tightly confined to sub-diffraction-limited gaps, enhancing fluorescence and revealing (ultra)strong light–matter coupling.^6−10^ These arise from the localized surface plasmons supported on metal nanostructures, which create tightly confined electromagnetic hot spots. The localized plasmons couple to radiative electronic transitions of any chromophore placed in the proximity, such as dye molecules or semiconductor quantum dots.^1−3,11^

Purcell effects and strong coupling are, however, not the only influence of plasmonic cavities. Recently, it has become clear that such tightly confined light can photodestabilize metal surfaces when coated with polarizable molecules.^12^ One effect is to pull single metal adatoms out of the facets surrounding nanoscale crevices, which locally enhances the optical field in a small volume of <1 nm^3^ (termed “picocavities”), and yields clear single-molecule surface-enhanced Raman scattering (SERS) that identifies individual vibrational bonds.^13,14^ Such ultraconfined light should enable single-molecule ultrastrong coupling, but dye emission remains puzzling from metallic nanogaps and has typically not been stable.^15−17^

Precise positioning of single emitters inside optical cavities is crucial for creating advanced photonic devices at the nanoscale. Aligning the emitting dipole with the peak optical field optimizes Purcell enhancements that increase emission rates, efficiency, and directionality.^18^ Numerous techniques have been investigated to place a single molecule into such plasmonic cavities, but important limitations center on the lack of control in fabricating the required structures. Because it remains difficult to precisely control the position and orientation of a specific number of emitting chromophores inside such optical cavities, the observation of single-molecule dye behaviors remains challenging.^19,20^

Using the addressability of deoxyribonucleic acid (DNA), we report a viable technique for controlling the precise arrangement of several quantum emitters in optical nanocavities lodged between two gold surfaces. Because of the specificity of the Watson–Crick base pairing and the inherent physical dimensions of DNA, functional structures and devices combining metallic and molecular components can be created with nanoscale precision.^21−26^ In DNA nanotechnology, a long circular strand of a viral genome is folded with a set of hundreds of customized short “staple strands”.^21,27,28^ These strands can be chemically modified with many species, including quantum emitters, proteins, small organic molecules, nanoparticles, and other moieties.^19,29−31^ Such DNA origami (DNAo) nanostructures thus function as molecular breadboards for the user-defined arrangement of different nanoscale components.^32−35^ Here, single emitters embedded in a DNA-built nanostructure are found to cause a light-driven rearrangement of the Au atoms in the surrounding metal facets. Instead of enhancing fluorescence, the plasmonic cavity changes the relaxation branching ratio to favor inelastic (Raman) scattering. The hundred-fold increased polarizability of the resonant dye enormously enhances the optical forces. Even though a theoretical model is currently not available for such complex systems, we observe that on-resonant dye molecules show strong interactions with the metal nanoparticles, implying that indeed, the polarizability of the molecules plays a key role in the generation of the observed optical forces. This dominates the emission signatures and requires a fundamental reconsideration of light–matter interactions at the nanoscale.

## DNAo as a Spacer for Precise Nanocavity Fabrication

**Figure 1:**
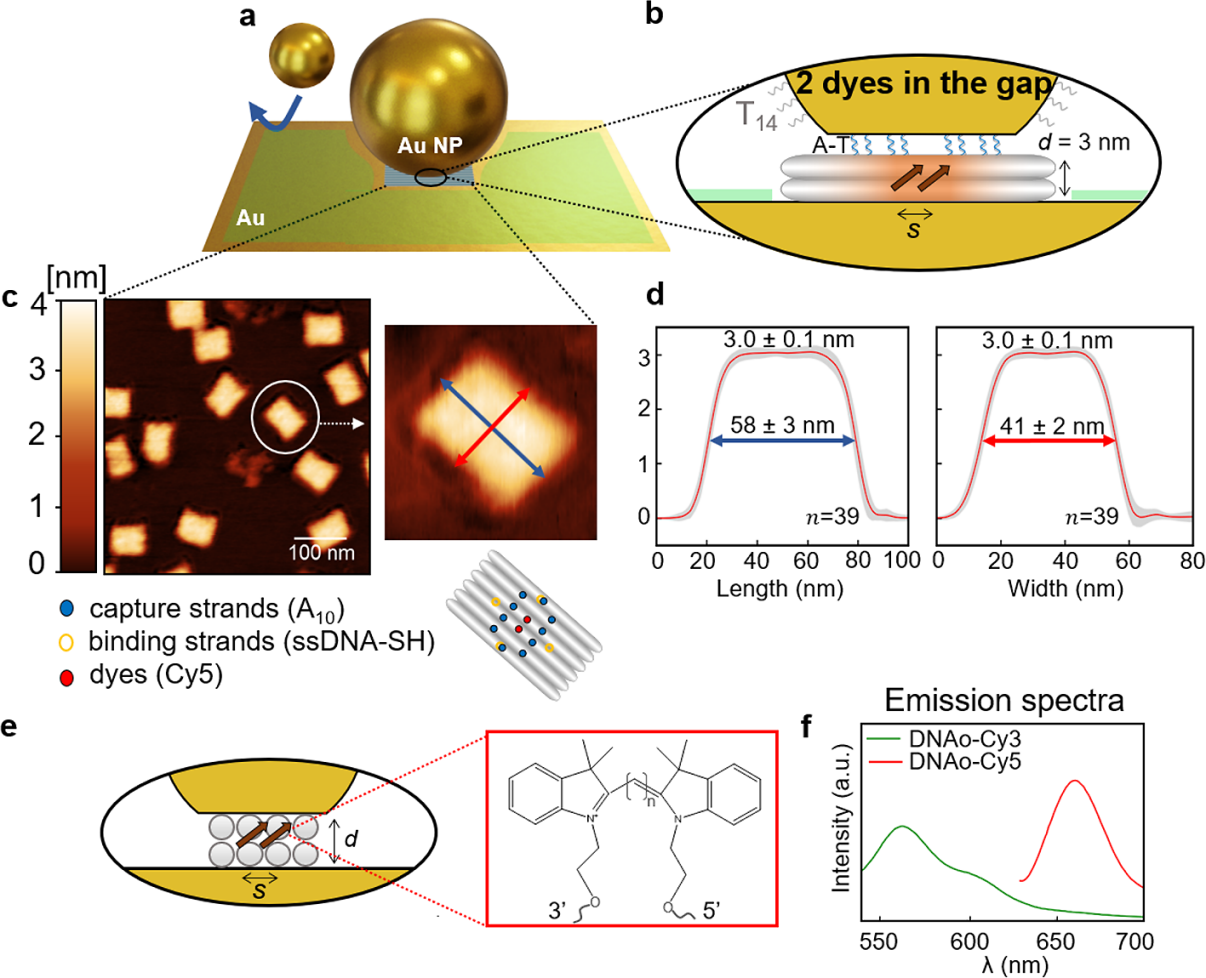
Assembly of NPoM cavities using DNA nanotechnology. (a and b) Gold nanoparticle with diameter *D* of 60 or 80 nm attached to the DNAo structure (gray) with thickness *d* of 3 nm in the NPoM geometry. Polyadenine strands from DNAo (blue in the inset) hybridize with polythymine strands (gray in the inset, T_14_) in the plasmonic gap via A-T binding. One or two dye molecules are placed in the plasmonic gap with a lateral separation *s* of 5 nm. A dodecanethiol monolayer (green) prevents nonspecific binding of AuNPs to the mirror in the absence of DNAo (Figure S5). (c) Atomic force microscope images of DNAo on mica confirm the correct folding of the DNA nanostructures. (d) Analysis of 39 nanostructures confirms dimensions of DNA positioners; red curves are average after registration along axes in panel c. (e) Chemical structure of cyanine dyes (*n* = 3 and 5 for Cy3 and Cy5 molecules, respectively) and (f) their emission in solution when incorporated into DNAo.

We exploit a robust nanocavity design based on the nanoparticle-on-mirror (NPoM) construct, because it is capable of scalable parallelized self-assembly of nearly identical nanocavities that can be spectroscopically characterized with ease. Using DNA origami solves the fundamental difficulty in placing single emitters in precise locations within each nanocavity. The NPoMs are fabricated using *D* = 60 or 80 nm diameter monodisperse citrate-capped superspherical gold nanoparticles [AuNPs (see Methods)] aligned on top of a template-stripped Au mirror by DNAo (Figure 1a). Larger particles are used for atomic force microscopy and dark-field measurements, while smaller particles are used for photoluminescence and surface-enhanced Raman (SERS) measurement to enhance the resonant tuning. Compared to previous designs,^29,36^ the DNA plate is redesigned to lie flat, covalently bind via thiol–Au bonds, and incorporate dyes that base-pair bind on both ends to better define orientations.^37^ The double-layer DNAo plate with a thickness *d* of ≃3 nm sets the height of the plasmonic cavity nanogap. Using this DNAo plate, one or two quantum emitters are used (Figure S3) and precisely positioned inside the gap between the two Au facets with a lateral separation *s* of 5 nm (Figure 1b). A hydrophobic coating of dodecanethiol on the Au mirror (green in Figure 1a,b) prevents any DNA-coated AuNPs from nonspecific binding to the metal, away from the DNAo plates.

Multiple atomic force microscope (AFM) images (Figure 1c,d) confirm the correct assembly and dimensions of the DNA nanostructures. The width, length, and thickness of 39 measured structures agree with expected literature values under dry conditions and with the planned design.^22^ The latter guarantees AuNP binding on one side only using several polyadenine chains (capture strands, blue in the insets of panels b and c of Figure 1) and binding to the Au substrate on the other side using short thiolated binding strands (inset of Figure 1c, exact DNA design and sequences in sections S1 and S2). Quantum emitters are purchased as internal modifications of a 32-nucleotide DNA strand (Figure 1e), which when assembled in the DNAo in solution gives their expected cyanine dye fluorescence spectra (Figure 1f).

**Figure 2:**
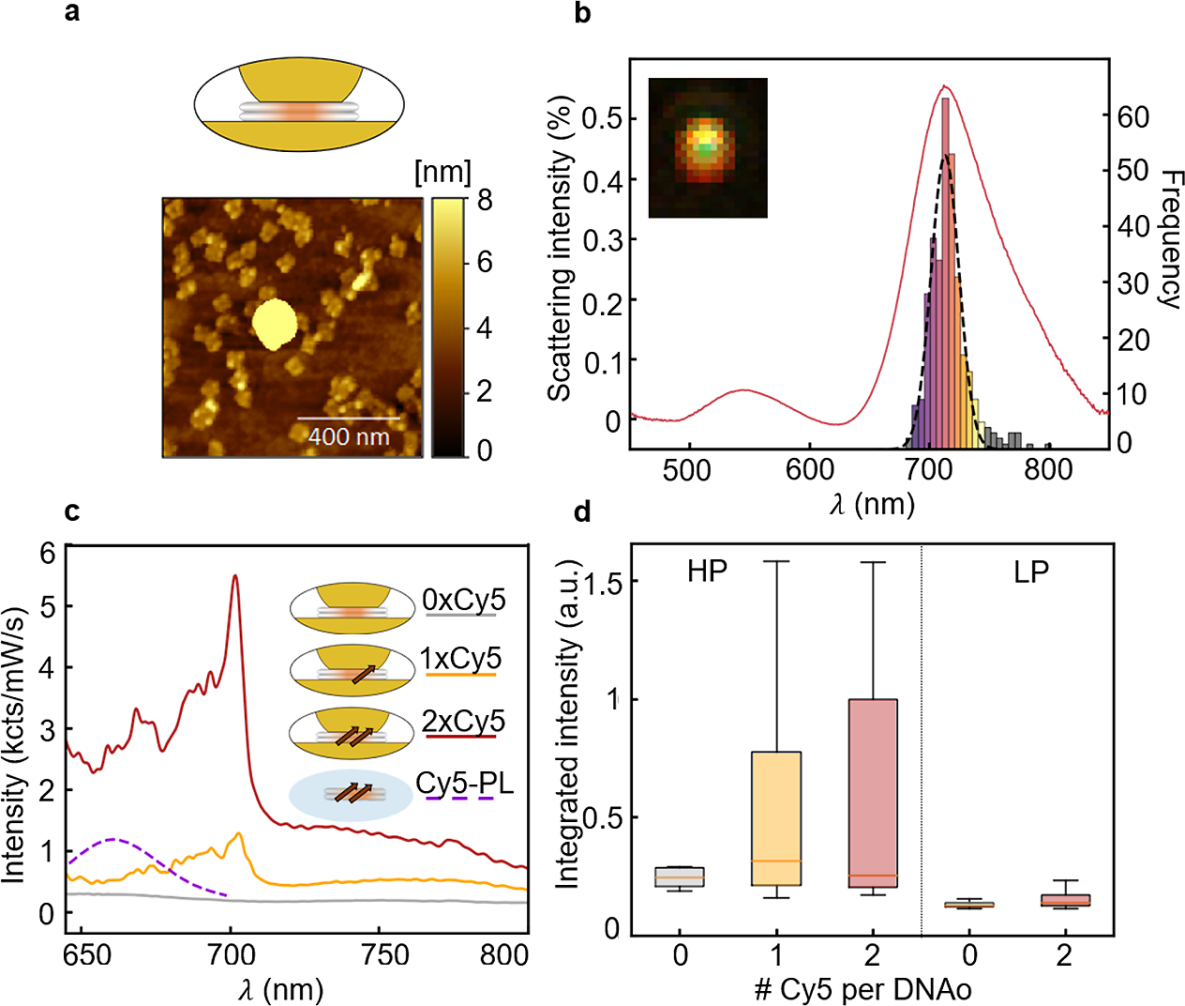
Characterization of DNAo NPoMs. (a) AFM shows 80 nm AuNP sitting on a DNAo-coated gold surface. Protruding DNAo at the edge around the AuNP suggests it is immobilized centrally on one DNAo. (b) Histogram of coupled mode wavelengths λ_10_ from dark-field scattering of >1000 NPoMs and average spectrum of the most common bin (orange). (c) Typical emission spectra of NPoMs with zero (gray), one (yellow), or two (red) Cy5 dyes within the DNAo plate. Solution emission of Cy5 in DNAo is shown as a purple dashed curve. (d) Statistics of emission intensities at high (HP) and low (LP) powers for zero-, one-, and two-Cy5 DNAo in NPoMs.

AFM imaging after NPoM assembly shows AuNPs on top of the DNAo-coated Au mirror (Figure 2a). It is not possible to directly visualize the binding between the nanoparticle and surface; however, the positioning of other DNAo in the vicinity suggests that specific binding by DNA strands is achieved (section S4). This is further confirmed by dark-field (DF) measurements, dynamic light scattering (DLS), and fluorescence measurements (sections S5–S7). Where not specified below, the washed samples are in a nominally dry state with water confined only within the DNAo.

Dark-field (DF) spectroscopy provides further insight into NPoM assembly. Specific plasmon modes exist inside each nanogap of a characteristic wavelength dependent on gap size and contents. DF optical images of dye-free NPoMs (inset of Figure 2b) show the expected circularly symmetric emission pattern from a *z*-oriented dipole, which gives high-angle emission from the dominant coupled plasmon mode (red ring), and normally directed emission from the transverse mode (green spot).^20^ Scattering spectra of >1000 individual NPoMs are recorded automatically to characterize the plasmon mode distribution across each sample (Figure 2b). The most prominent modes are the lowest-energy coupled mode (λ_10_) at ∼710 nm and the transverse mode at ∼530 nm. Sorting the frequency distribution of λ_10_ allows extraction of the representative scattering spectra (Figure 2b, orange curve) averaged within the most common histogram bin.^38,39^

The plasmonic gap size is set by the DNAo spacer, whose dimensions are consistent with the literature.^40^ Despite being highly sensitive to the nanoparticle morphology, including their facet size, diameter, and contact position on the DNA nanostructure, the narrow size of the λ_10_ distribution shows that most NPoMs have identical gaps and contents. Electromagnetic modeling implies a gap size *d* of 4 nm, a nanoparticle diameter *D* of 80 nm, and a gap refractive index of 1.5.^41−43^ Given the measured height of DNAo (Figure 1c), a gap of 4 nm takes into account the extra distance added by the adenine-thymine binding of a AuNP to DNAo and possible effects of compression of the metal particle on the softer material. The electromagnetic environment of the dyes is thus well-defined, with an expected Purcell factor of >100. Selection of the NP diameter here gives good overlap between Cy5 photoluminescence (PL) and the λ_10_ coupled plasmon.

Light emission from DNAo-scaffolded NPoMs is studied by resonantly pumping individual NPoMs at 633 nm. However, instead of straightforward plasmon enhancement of the PL, the emission acquires strong contributions from surface-enhanced resonant Raman scattering (SERRS) of the dye (Figure 2c, orange and red curves). This can be seen from the sharp peaks, which vanish (along with a large fraction of the background) when no dye is included in the construct (Figure 2c, gray, <0.5 kcts mW^–1^ s^–1^), although their DF spectra remain unchanged (Figure S12), showing that there is no modification of gap size or coupling. By studying more than 300 NPoMs, we found statistics on light emission from gaps with zero, one, and two Cy5 dyes provide further confirmation of this behavior (Figure 2d). At both high powers (HP, 0.7 mW/μm^2^) and low powers (LP, 0.2 mW/μm^2^), systems with one or two quantum emitters show significantly stronger light emission than systems with no Cy5.

**Figure 3:**
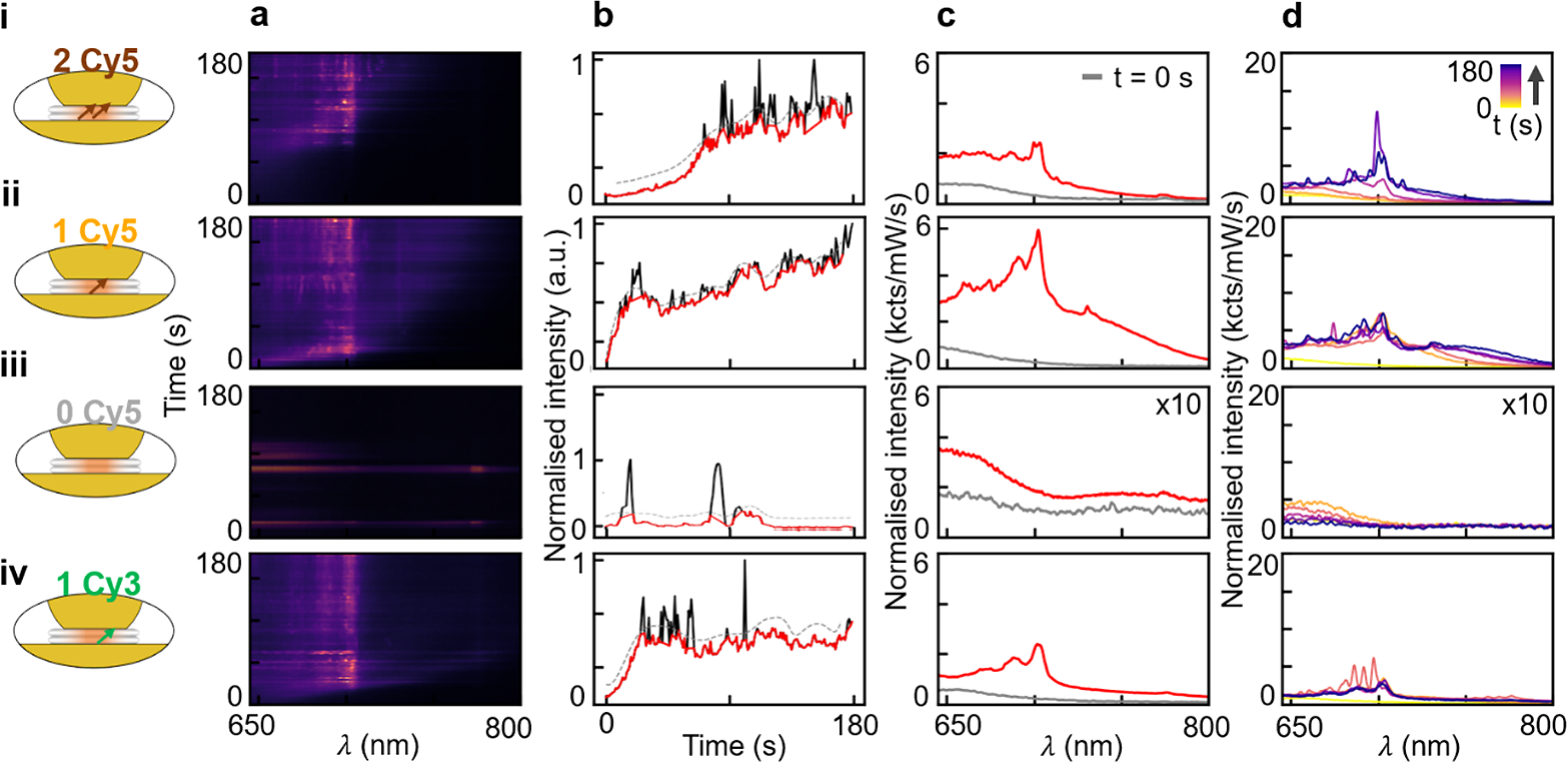
Dynamics of NPoM emission with (i) two, (ii) one, or (iii) zero Cy5 dyes or (iv) one Cy3 dye (schematics shown at the left). (a) Time-dependent emission spectra were recorded over 180 s with a 633 nm pump laser. (b) Spectrally integrated emission intensity vs time. Dotted lines show the shifted running average used to distinguish continuous emission (red) from spikes (black). (c) Average emission from spectra without spikes seen in panel b (red) and spectra at time zero (gray). (d) Emission spectra every 30 s (color denotes time).

The spectra when one or two quantum emitters are positioned inside the NPoMs (Figure 3i,ii,iv) appear very different from their solution emission (purple dashed line in Figure 2c). Representative spectra from >200 individual NPoMs on each sample show the dramatic increase in intensity when one (orange) or two (red) Cy5 molecules are present (Figure 2c). The emission (including SERRS) increases consistently with the number of Cy5 molecules, and spectral features appear in the range of 640–700 nm, very unlike the solution PL. Moreover, the peaks in the range of 680–710 nm associated with Cy5 molecules change over time, suggesting time-dependent interactions among the emitter, the metal surfaces, and their chemical environment, induced by the light.

To confirm this hypothesis, time scans of successive spectra from individual NPoMs containing two, one, or zero molecules of Cy5 within DNAo (panel i, ii, or iii, respectively, of Figure 3) are collected using 633 nm laser illumination at 0.7 mW/μm^2^ for 180 s (emission normalized to laser power, integration time of 1 s). Typically, the emission maps (Figure 3a) increase over time and shift weight to longer wavelengths. Note the emission without dye molecules (Figure 3a,iii) is much weaker and shows occasional “flare” events characteristic of Au facet transient defects, as well as from DNA bases.^44,45^

To better resolve the dynamics, spectra integrated from 680 to 800 nm are tracked over time (Figure 3b, normalized). Monotonic growth in integrated intensity is mostly observed along with intense sporadic spikes, which can be separated out (black vs red curves). We first concentrate on the underlying background emission (red curves) and average these to obtain their characteristic spectral shapes (Figure 3c), as well as at different points in time (Figure 3d). In this way, anomalous high-intensity spikes do not distort the spectra.

Without dyes (Figure 3b,iii), emission is again weak and featureless and decreases with time. This implies that the chemical environment of the DNAo is not changing, nor is it rearranging or contracting the nanogap by excluding water (as observed at higher laser powers in DNAo-bound dimer samples).^46^ By contrast, even a single dye molecule shows completely different emission dynamics, increasing with time and showing distinct and strong emission peaks of >20 kcts mW^–1^ s^–1^ (Figure 3b–d,ii). The fact that this extreme influence on NPoMs is from only a single chromophore is confirmed by solution PL (without NPoMs), which shows that indeed, only single Cy5 dyes integrate into each DNAo (section S3). The progressive emergence of spectral features over time suggests that the dye molecule in NPoMs interacts with the local DNA, metal surfaces, or ionic environment. Although their dynamics varies, collected intensities for >400 individual NPoMs containing zero to two Cy5 molecules show systematic effects (Figure 3d) directly related to the presence of this two-level quantum emitter within the plasmonic cavity.

**Figure 4:**
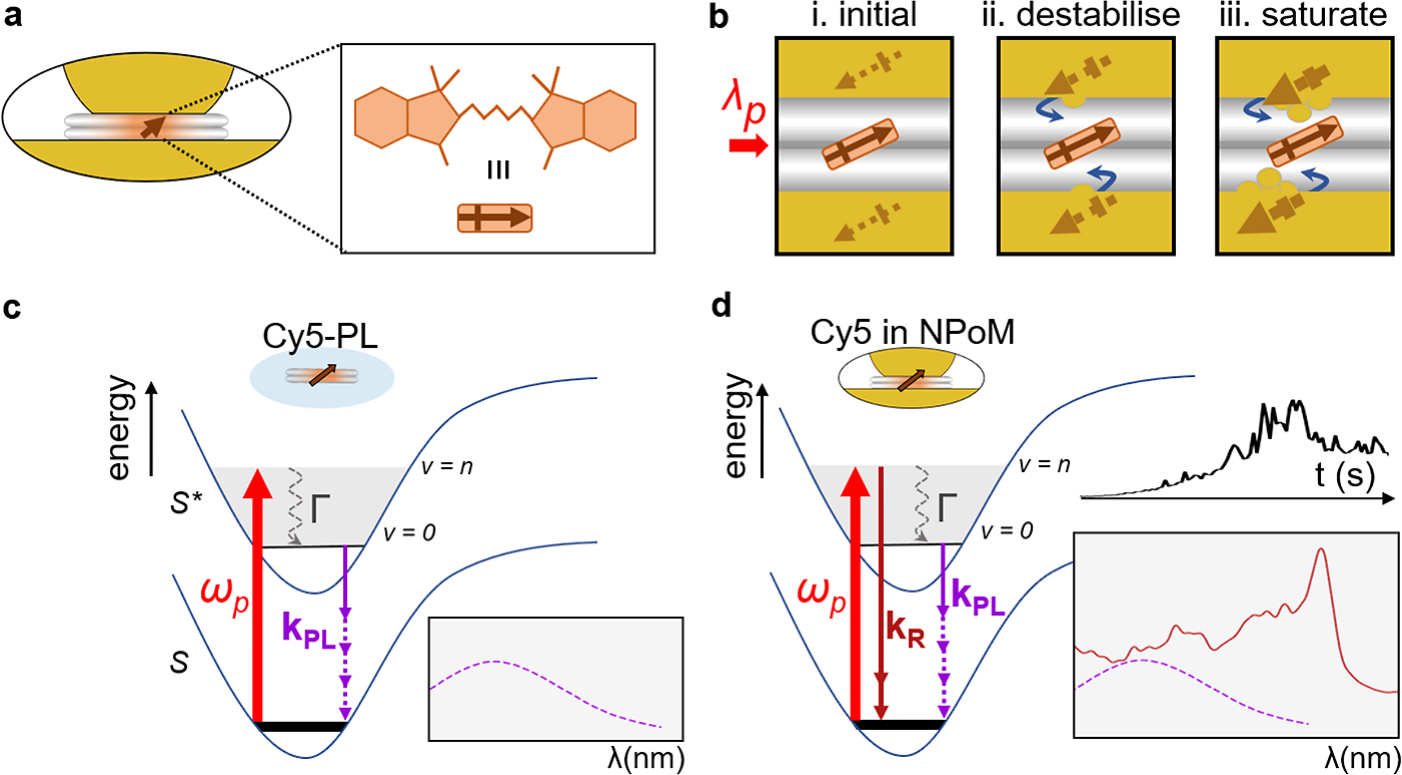
Proposed model of dye–metal interactions in DNAo-scaffolded plasmonic cavities. (a) Schematic NPoM containing a Cy5 (dark red) within DNAo. (b) A light-driven (red solid arrow; λ_p_ = 633 nm) extended molecular dipole induces local plasmonic dipoles, creating strong dye–metal dipole–dipole attraction (blue arrows). From (i) the initial state, (ii) this gradually pulls gold atoms from facets toward the molecule (destabilization phase), eventually reaching (iii) saturation. (c and d) Jablonski diagrams showing the PL of Cy5 and the proposed plasmonic mechanism, respectively, in which SERRS processes (*k*_R_) become faster than fluorescence because of extreme Purcell enhancements.

The effects observed here vary only quantitatively with the laser intensity. At lower powers, similar spectra are observed (Figure S8). Further understanding is gained by changing the dye. Although Cy3 emits at shorter wavelengths in solution [shifted by 100 nm (Figure 1f)], using one Cy3 within the DNAo–NPoM constructs does not greatly change the shape of the spectrum compared to that with Cy5 (Figure 3,iv), showing only a smaller shift in emission (<20 nm). Alternative conjugated dyes similarly do not modify the emission spectrum (Figure S9). As a result, we conclude the NPoM emission spectrum is not simply Purcell-enhanced fluorescence but instead is heavily modified by the plasmonic environment. Our proposed mechanism (Figure 4) suggests that the branching ratio between PL and SERRS [which in solution allows the observation of typical dye fluorescence (Figure 4c)] in NPoMs is heavily weighted toward the latter because the speed of vibrational/rotational relaxation from higher vibrational states (Kasha’s rule) in the excited manifold (Γ) is now too slow to compete with the Purcell acceleration in radiative emission *k*_R_ of the SERRS and Rayleigh scattering (Figure 4d). Dye fluorescence lifetimes of 2–4 ns^47,48^ have been shown to be Purcell-accelerated by >4000 in such nanogaps from enhanced fields *E*/*E*_0_ of ∼100. Thus, they can indeed become faster than the typical 50–100 fs vibrational relaxation in the excited state manifold^49^ and similar to the photon lifetime in the nanocavity (2π*Q*/ω_c_ ∼ 50 fs).^36^ This accounts for the shape of the observed spectra and their dependence on the vibrational fingerprint of the dye and not its emission spectra. It also accounts for why bleaching is rarely observed in this system because the molecules spend minimal time in their excited state.

However, this does not explain the slow increase in emission or the intense spikes, which imply morphological changes. When the laser is turned off, the emission does not return to the initial *t* = 0 level (Figure S10), confirming long-lived changes are induced. We discount light-induced ionic effects on the dye as no effect is seen upon washing the samples with different salt concentrations or detecting the emission in the wet state, which excludes any influence of drying processes (Figure S11). Two possibilities are suggested for the action of these light-driven dyes. Either they change in position and/or orientation, or they induce changes in the Au atoms several nanometers above and below. Recent results show optical forces from nonresonant molecules can be 1000-fold larger than expected and pull out individual Au atoms from the facets, implicating the latter effect.^50^

As a result, we suggest that irradiated dye molecules generate an optical “nanotractor beam” that sucks Au atoms out of the facet. Compared to nonresonant molecules, the enhancement of susceptibility from a two-level system driven near resonance massively increases the already large forces (set by absorption *Q*_α_ ∼ λ/Δλ ≃ 10, where λ is the absorption wavelength and Δλ the bandwidth). Although the nanogap here is 4 nm, the forces are extremely sensitive to the distance between polarization dipoles and metal surface. In our model (which is developed from ref (50)), nanocavity optical fields [oriented normal to the facet in such thin nanogaps (*E*/*E*_0_ ∼ 100)] polarize the extended molecular dipole (orange). A possible preliminary stage is the light-induced rotation of the dipole parallel to the field (a type of near-field optical Fredericks transition).^51^ Its resonant dipolar susceptibility is then massively enhanced by the corresponding multiple-image charges in the plasmonic metal facets above and below (Figure 4b). This induces sufficiently strong dipole–dipole forces on the closest metal atoms to pull them closer. This in turn increases the resonantly induced dipoles, thus increasing the forces that then attract even more Au atoms.^50^ The stochastic nature of this process as well as the instability of such a few atom Au tip lead to the observed fluctuations.

The destabilization of the Au facets by the oriented high-susceptibility electronic transition is thus responsible for changing the light emission. The initial state (Figure 4b,i) positions the quantum emitters at their designed location inside the DNAo, so the observed spectrum is dominated by a weak background contribution arising from the emission of free carriers in the Au [either metal photoluminescence or electronic Raman scattering (ERS)]. As the dye nanotractor beam pulls Au atoms closer, extra field enhancement *F* ∼ 5 at the tip due to the lightning rod effect, which spectrally tunes with picocavity morphology,^13^ enhances the SERRS ∝ *F*^4^ ∼ 600 and introduces new coordination bonds between Au and conjugated carbon chains that give the strong peaks that emerge (Figure 4d).

Compared to picocavities seen from single adatoms in nanocavities and crevices, the much stronger resonant electronic transition here leads to comprehensive restructuring of the facet to bring Au nanotips into contact with the dye, presumably forcing their way through the surrounding DNA (Figure 4b,iii) We believe that such effects are present in many systems with electronic transitions inside nanocavities, but these have been hard to study in the past. Here the robust rigid nanostructure with deterministic digital control of quantum emitter placement gives convincing systematic evidence for such optomolecularly induced forces. Because significant efforts are underway to study the ultimate strong coupling with emitters inside ultrasmall optical cavities, the implications of this work are that opto-mechanical forces are non-negligible and dominate the evolution of the system. At the same time, a full understanding (which demands a currently intractable theoretical model combining quantum molecular and electromagnetic plasmonic components) should lead to control and exploitation of such forces.

We show that DNA origami can be used to position single-quantum emitters in a plasmonic cavity between a gold nanoparticle and a gold mirror. Given the robustness of the DNA origami technique, and the refined protocol of immobilization developed in this work, precise positioning of AuNPs on DNAo to create homogeneous plasmonic hot spots is achieved. As a result, the interaction of the tightly confined light of quantum emitters with atoms from the metal surfaces can be studied. Only when quantum emitters are present within the DNAo nanostructures do unusual spectral signatures emerge that evidence resonant SERRS. Along with systematic controls with and without different dye molecules, these suggest the development of extremely strong optical forces forming nanotractor beams. These can pull gold atoms away from the metal surface and enhance its electronic Raman and picocavity SERRS emission.^12,13^ Implications of such light-destabilized molecule–metal interfaces range from photocatalysis to nanoassembly and quantum control.^16^

## Methods

### DNA Origami Folding and Purification

A single-stranded viral DNA scaffold of 7249 bases isolated from the M13mp19 derivative is folded into rectangular DNA origami structures in 12 mM MgCl_2_ and 1× TE buffer and purified from excess staples using centrifugal filtration. Further details about the structural design and folding can be found in section S13a.

### Functionalization of Gold Nanoparticles

AuNPs are functionalized with an excess of polythymine strands carrying a dithiol group on their 5′ end side. The protocol is described in section S13b.

### NPoM Assembly

DNA nanostructures are immobilized on a template-stripped gold film via their thiolated strands. A hydrophobic layer of dodecanethiol in ethanol is used to passivate the free Au surface. DNA-functionalized AuNPs are then drop cast on the DNAo and allowed to hybridize (section S13c).

### AFM Imaging of DNA Origami and NPoM

AFM images were acquired on an MFP-3D AFM system (Asylum/Oxford Instruments) using OTESPA silicon probes with a visible apex tip in tapping mode. Background correction and analysis were performed using the software Gwyddion. DNAo structures (2 μL, 2 nM) were drop cast and incubated on a mica surface (Agar Scientific) for 60 s. Excess structures were rinsed away with Milli-Q water, and the surface was dried with a stream of nitrogen for 30 s prior to imaging. To image NPoMs, the protocol for immobilization described earlier was used.

### Single Nanoparticle DF and SERS Measurements

Both DF and SERS spectra are recorded on a home-built confocal Raman microscope. The setup features and details are described in section S13e.

## Data Availability

Source data can be found at DOI link: https://doi.org/10.17863/CAM.97152.

## Supporting Information Available

DNAo design and staples (sections S1 and S2), fluorescence emission from dye-labeled DNAo (section S3), AFM images with different scale bars (section S4), dark-field measurements to control AuNP immobilization on DNAo (section S5), DLS measurements of DNA-functionalized AuNP (section S6), fluorescence quenching by AuNPs (section S7), light emission at lower power (section S8), ATTO647N (section S9), long-lived state (section S10), wet NPoM (section S11), dark field before and after laser illumination (section S12), and methods and materials (section S13) (PDF)
