# Supplementary material for: Amplified Plasmonic Forces from DNA Origami-Scaffolded Single Dyes in Nanogaps
Source: Nano Lett. 2023 Jun 26;23(13):5959–66. doi: 10.1021/acs.nanolett.3c01016 (PMC10347698; doi:10.1021/acs.nanolett.3c01016)
Supplement: Supplementary file 1 — nl3c01016_si_001.pdf [file nl3c01016_si_001.pdf]

Supplementary Information

Amplified plasmonic forces from DNA-origami scaffolded single dyes in nanogaps

Sara Rocchetti[1], Alexander Ohmann[1], Rohit Chikkaraddy[1,2], Gyeongwon Kang[1], Ulrich F. Keyser[1*], Jeremy J. Baumberg[1*]

[1] Nanophotonics Centre, Department of Physics, Cavendish Laboratory, University of Cambridge, Cambridge, CB3 0HE, England, UK

[2] School of Physics and Astronomy, University of Birmingham, Edgbaston, Birmingham, B15 2TT, England UK

* e-mail: jjb12@cam.ac.uk and ufk20@cam.ac.uk

S1. DNAo design and staples

a

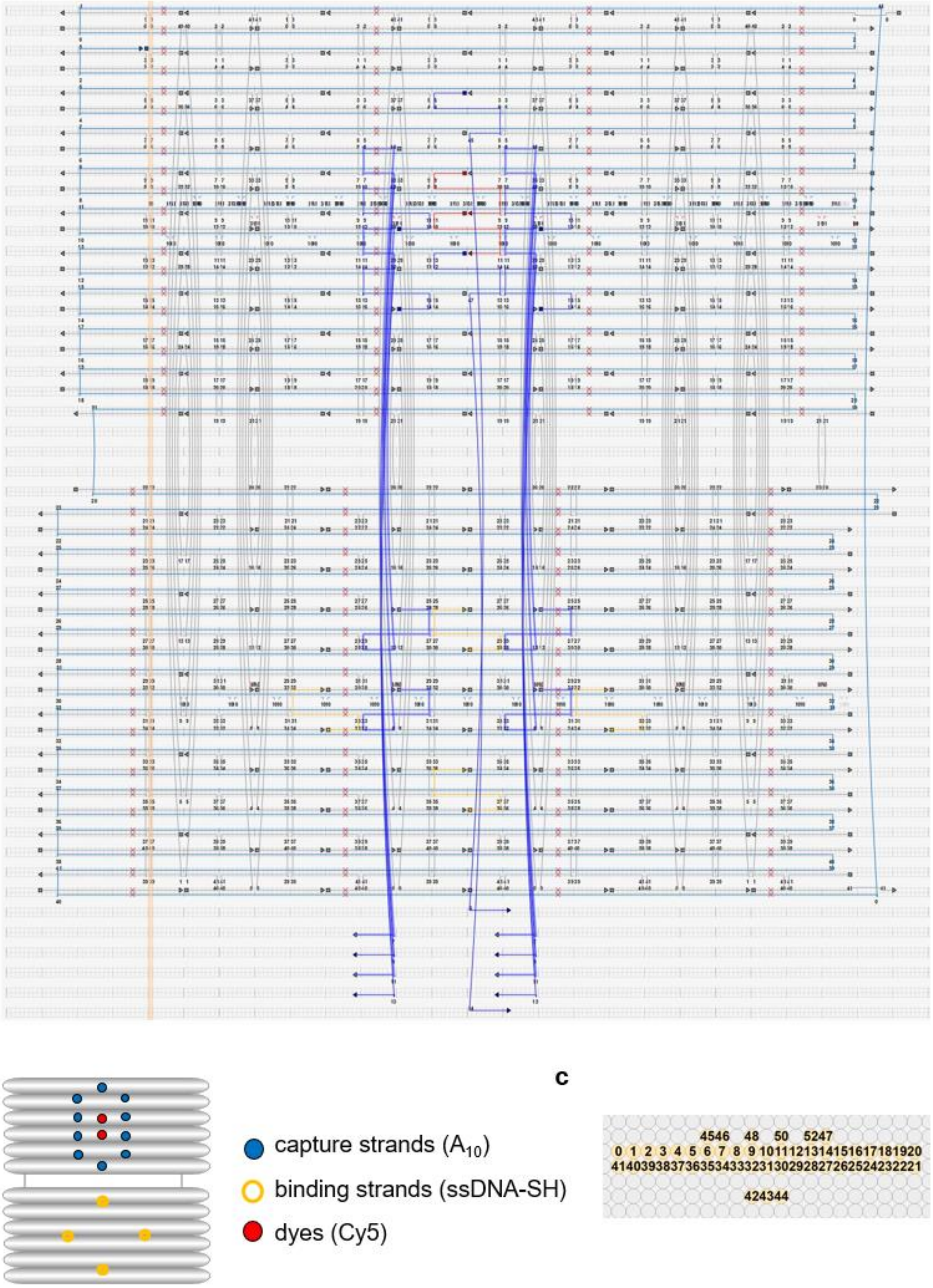

Figure S1. DNA origami structure. a. Design of the DNA nanostructure from the software caDNAno. The 7249 nucleotides long scaffold (light blue) is folded into a two-layer plate with the aid of customised short staple strands (grey). Highlighted staple strands indicate functionalised ssDNA as depicted in **b.** Blue colour refers to capture strands (poly-A nucleotides) on the top layer of the DNA plate, yellow colour refers to thiolated nucleotides on the bottom layer and red colour refers to dye-labelled strands. **c.** 2D layout of helices: 0-20 indicate top layer and 21-41 indicate bottom layer.

S2. DNAo staple strands

Unmodified oligonucleotides

Start	End	Sequence
0[103]	2[104]	AGTGTAAATTTCACCAGTGAGACGGTGTTGTT
0[135]	2[136]	ATTCCACCCTTCACCGCCTGGCAAATCAAA
0[167]	38[168]	TGTTTCCTCAAGCGGTTTGGTGTACCGTGGGAACAAACGGCTATC
0[201]	0[168]	CCCTGAATTCGTAATCATGGTCATAGC
0[39]	38[40]	CCGCTTTAATGAATAAGCGCCAAATTTTTGTTAAATCATTGTATAA
0[71]	2[72]	CTAACTCATTTGCGTATTGGGCGCATTAAAGA
1[12]	0[12]	CCCTTCGTGCCAGCTGCATTCCAGTCGGGAAACCTGCCCT
1[120]	41[135]	CTGATTGCACAACATATCACGACGTTGTAAA
1[152]	41[167]	AGTTGCAGGTGTGAAAAGCTTGCATGCCTGCA
1[56]	41[71]	AGAGGCGGCATTAATTCAGCTGGCGAAAGGGG
1[88]	41[103]	GTTTTTCTGCCTGGGGGATTAAGTTGGGTAAC
10[135]	12[136]	AAATTATATTTAACAATTTCATCTTAGAAT
10[167]	12[168]	GAATAATGAATGGAAACAGTACATTTCTGTAA
10[195]	9[195]	CCCTCTGATTGTTTGGATTTTCATCAATATAATCCCCT
10[39]	12[40]	GAGAAACAAGTTACAAAATCGCTTTTTAAC
10[71]	12[72]	TGAATATACATTTCAATTACCTGAATTTATCA
11[12]	10[12]	CCCTGATTGCTTTGAATACCAATAACGGATTCGCCTCCCT
11[152]	9[151]	CCTTTTTTGAAGGGTTAGAACCTATCATCATA
11[56]	9[55]	GAATTATTCAGTAACAGTACCTTTTATTAAAT
12[135]	14[136]	CCTTGAAAAATACCGACCGTGTTATAAAGC
12[167]	26[168]	ATCGTCGCAATAAGAATTTGCGGGCGATATATTCGGTCGACAGACA
12[195]	11[195]	CCCTAGTGAATAACCTTGCAAATCAATATATGTGCCCT
12[39]	26[40]	CTCCGGCTGATGCATGCCACTAAAAAAAGGCTCCAAAAAAAGGAAC
12[71]	14[72]	AAATCATACGAGAAAACTTTTTCATGTAATTT
13[12]	12[12]	CCCTAACTATATGTAAATGCTTAGGTTGGGTTATATCCCT
13[120]	31[119]	ATGGTTTGAACATAGCGTCGAAAAGGCTGGCTGACCTTAGGACGTT
13[152]	31[151]	AGGCGTTATATTAATTAGCCGGAAGGACAGATGAACGGTGAAACGAAC
13[56]	31[55]	AAAGAACGGGTCTGAGAGCGATTAACGTAACAAAGCTGCTTTTCAACT
13[88]	31[87]	TTTAGTTAAAGAGTCAACGGAGATGACAAGAACCGGATATGCGATTTT
14[103]	16[104]	CGCCATATAAGTCCTGAACAAGAACAATAGCA
14[135]	16[136]	CAACGCTTAATTTACGAGCATGTTATTTTC
14[167]	16[168]	ATGCGTTAATCGGCTGTCTTTCCTTACCGCAC
14[195]	13[195]	CCCTAGAAAAAGCCTGTTTGGAATCATAATTACTCCCT
14[39]	16[40]	AATATAAGACGACGACAATAAACGAACCTC
14[71]	16[72]	AGGCAGAGTGCAGAACGCGCCTGTATCCGGTA
15[12]	14[12]	CCCTAAAGTAATTCTGTCCAAGTACCGACAAAAGGTCCCT
15[152]	13[151]	AATCAATATACAAATTCTTACCAGGATAAATA
15[56]	13[55]	TCAGCTAAGCATTTTCGAGCCAGTCGCAAGAC
16[103]	18[104]	AGCAAATCTCCAGAGCCTAATTTGAAAGTCAG
16[135]	18[136]	ATCGTAGCAGCCATATTATTTATAGACGGG
16[167]	22[168]	TCATCGAGAAACGATTACAGTTAAAAGTTTTAACGGGGTTTAGCGT
16[195]	15[195]	CCCTGGGTATTAAACCAAGTATCATTCCAAGAACCCCT

16[39]	22[40]	CCGACTTGTTGCTACCGTCGAGATTGGCCTTGATATTCAGAACCAC
16[71]	18[72]	TTCTAAGACCTGAATCTTACCAACGAGAGATA
17[12]	16[12]	CCCTCCTTAAATCAAGATTAGCGGGAGGTTTTGAAGCCCT
17[120]	27[119]	AAAATAAAGAATCATTGCAAGCCTTCCAGACGTTAGTAACCGATAG
17[152]	27[151]	CAAATAAGAACAAGCAACTGAGTTAGTTAGCGTAACGATCATCGCCCA
17[56]	27[55]	AATTTTATACGCGAGGCCCTCAGAAGCGGAGTGAGAATAGGGAGCCTT
17[88]	27[87]	AGCGTCTTAGATATAGCCTCAGAGGGGATTTTGCTAAACAGCTTTCGA
18[103]	20[104]	AGGGTAATCGCAATAATAACGGAAACCAGCGC
18[135]	20[136]	AGAATTACATGATTAAGACTCCTGTCACAA
18[167]	20[168]	TAACATAATAGCAAACGTAGAAAAAAACGCAA
18[195]	17[195]	CCCTAATAGCAGCCTTTACAACGTCAAAAATGAACCCT
18[39]	20[40]	GAGCAAGAGCCCTTTTTAAGAAGAAATTAT
18[71]	20[72]	ACCCACAAGAACAAAGTTACCAGAACCGATTG
19[12]	18[12]	CCCTATAGCTATCTTACCGAAAACAATGAAATAGCACCCT
19[120]	17[119]	AGAACTGGACTGAACACCCTGAACCCAGTTAC
19[152]	17[151]	CAGTATGTAAACAGGGAAGCGCATTCCCAATC
19[56]	17[55]	AGATAGCCGAATTGAGTTAAGCCCCCAGCTAC
19[88]	17[87]	CGAGGAAATGAGCGCTAATATCAGCTAACG
2[103]	4[104]	CCAGTTTGCCGATTTAGAGCTTGAGAGCACGT
2[135]	4[136]	AGAATAGACGTGGCGAGAAAGGGCTACAGG
2[167]	4[168]	TCCGAAATAGGAGCGGGCGCTAGGGTAACCAC
2[195]	1[195]	CCCTCGAAAATCCTGTTTGGTTTGCCCCAGCAGGCCCT
2[39]	4[40]	GTCTATCAAATCAAGTTTTTTGAGGGATTT
2[71]	4[72]	ACGTGGACAAGCACTAAATCGGAATCAGAGCG
20[103]	23[87]	CAAAGACAAAAGGGCGCAAGGCCGCCCTCAGAACCGCCACTGGAAAGC
20[135]	23[119]	TCAATAGAAAATTCAATAGCAGACCACCGGAACCGCCGTAAGCGT
20[167]	23[151]	AGACACCACGGAATAACAAGTTTGTTTTCATAATCAAAATGGAGTGTA
20[195]	21[200]	CCCTGGCAACATGTTTTCATCGGCACCCT
20[39]	20[15]	TCATTAAAGGTGAATTATCACCCT
20[71]	23[55]	AGGGAGGGAAGGTAAAGCAAAATCCCTCAGAGCCGCCACCACAAACAA
21[104]	19[119]	ACCAATGAAACCATCGTATGGTTTTACCCAAA
21[136]	19[151]	TCAGTAGCGACAGAATGTTTATTTTTATTACG
21[15]	22[7]	CCCTCCGTCACCGACTCCGCCGCCAGCATTGACAGGCCCT
21[176]	19[195]	TGTAGCGCATAAAAGTACATACATAAAGGTCCCT
21[72]	19[87]	CACCATTACCATTAGACATTCAAGGAAAC
22[167]	21[175]	TTGCCATCCCTTTAGCGTCAGAC
22[200]	23[190]	CCCTTTTTCGGTCATAGCCCCCTTACAGTGCCTTGACCCT
22[39]	19[55]	CACCAGAGTGAGCCATTTGGGAATTAGAGCCATATTGACGAAGTAAGC
23[104]	21[103]	CCGTTCCATCCCTCAGAGCCGCCAGAAACGTC
23[120]	15[119]	CATACATGAAACATGAAAGTATCAGGGATAACCGCGCCAAATAATA
23[136]	21[135]	ATGATACACACCGGAACCAGAGCCCACCGTAA
23[152]	15[151]	CTGGTAATTGCCCCCTGCCTATTTACCGTAACAGCCGTTTTAGAAACC
23[56]	15[55]	ATAAATCCTGCTCAGTACCAGGCGTACCGCCACGTTTTAGCAACATGT
23[7]	24[7]	CCCTAGGTTGAGGCAGGTCAGACGAGGGTTGATATAAGTATAGCCCCT
23[72]	21[71]	GCCAGAACCTCAGAGCCACCACACCAGTAG
23[88]	15[87]	GCAGTCTCCTCAAGAGAAGGATTAACCGCCACAAGGCTTTTATCAA

24[190]	25[190]	CCCTGTAACAGTGCCACTACAACGCCCCCT
25[104]	23[103]	CTCATTTTTAAGAGGCTGAGACTCTGAATTTA
25[136]	23[135]	ACCCATGTCGGAACCTATTATTCTGGCTTTTG
25[7]	26[7]	CCCTCCGGAATAGGTGTATCACCGGAATTGCGAATAATAATTTTCCCT
25[72]	23[71]	CCTCAGAGGATTAGCGGGGTTTTCATTAAA
26[167]	18[168]	GCCCTCATTCGTCACCAGTACAACGTATAATTTTGTTTAGAGAGAA
26[190]	27[190]	CCCTTGTAGCATTCCCTGAGGCTTGCCCCT
26[39]	18[40]	AACTAAAGTACTCAGGAGGTTTAGGATAAGTGTTTTGCACAATAATAA
27[104]	25[103]	AGCTTGATAATGAATTTTCTGTATCCACCACC
27[136]	25[135]	CAACAACCTAAAGTTTTGTCGTCTCAATAGGA
27[152]	11[151]	CGCATAACATCGTCACCCTCAGCATGTTACTTAATTTTCCTTGAATTA
27[56]	11[55]	TAATTGTACCATTAAACGGGTAAATGACCCCCAGACTACCGCAGAGGC
27[7]	28[7]	CCCTTTCACGTTGAAAATCTCCAACGAAGGCACCAACCTAAAACCCCT
27[72]	25[71]	TCAGCTTACTTTCAACAGTTTCACCGCCAC
28[190]	29[190]	CCCTAGGGAGTTAAACAATCATAAGGCCCT
29[136]	27[135]	CTGCTCCAGCGAAAGACAGCATCGGACAATGA
29[7]	30[7]	CCCTGAAAGAGGCAAAAGAATACACTTGCCCTGACGAGAAACACCCCT
29[72]	27[71]	GCGAAACTTCATGAGGAAGTTTTCGGTTTA
3[12]	2[12]	CCCTACGTGAACCATCACCCAGGGCGATGGCCCACTCCCT
3[120]	1[119]	GCCGGCGACCCGAGATAGGGTTGAGGCAACAG
3[152]	1[151]	AAAGCGAACGGCAAAATCCCTTATCCTGAGAG
3[56]	1[55]	GTGCCGTATCCAACGTCAAAGGGCGCGCGGGG
3[88]	1[87]	GGGAGCCCGAACAAGAGTCCACTCAGGGTG
30[167]	14[168]	TTGAAAGACGAGGCGCAGACGGTGGCCGCTTAAACACCAGTATCAT
30[190]	31[190]	CCCTGAACCGAACTGTAGAAAGATTCCCCT
30[39]	14[40]	GAATAAGGCTAAAACACTCATCTTATACGTAAAATCCAATAATAAGAG
31[104]	29[103]	TACCAGTCCATCAAGAGTAATCTTTTGTATCA
31[136]	29[135]	CGTTAATATACAGACCAGGCGCATTCCGCGAC
31[152]	7[151]	TAACGGAATTCAACTAATGCAGATACTATTATTCAATCAACACCGCCT
31[56]	7[55]	TTAATCATCCAAAATAGCGAGAGGAATCGTCATAGAGCCGAATGCGCG
31[7]	32[7]	CCCTCAGAACGAGTAGTAAATTGGTTTGCCAGAGGGGGTAATAGCCCT
31[72]	29[71]	ACCTTATTCATTACCCAAATCATACCAAGC
32[190]	33[190]	CCCTATCAGTTGAGATCAAAAAGATTCCCT
33[104]	31[103]	CGAGAATGTAAGAGCAACACTATCGCTCATTA
33[7]	34[7]	CCCTTAAAATGTTTAGACTGGATAATTGCTGAATATAATGCTGTCCCT
34[167]	10[168]	CTTCAAATGCAAAGCGGATTGCATTTAGGAGATGGCAAATACTTCT
34[190]	35[190]	CCCTAAGAGGAAGCCGCGAGCTGAAACCCT
34[39]	10[40]	AGAGCTTAGCGTCCAATACTGCGGCTTTTGCAACAACTCGTACATCGG
35[104]	33[103]	ATTTAGTTTCCAACAGGTCAGGATTCAGAAAA
35[120]	3[119]	AGATACAAGGCAAAGAATTAGCCAAATCACGCTTTGACCGGGGAAA
35[136]	33[135]	ATGGTCAAAGCTTCAAAGCGAACCTCAGGTCT
35[152]	3[151]	TTAGCTATGTAGCATTAACATCCACTAGCTGAAATGCGCCAAGGGAAG
35[56]	3[55]	AGTTTCATCAAAAACATTATGACCAAATGCAACCGATTAAGGGTCGAG
35[7]	36[7]	CCCTAGCTCAACATGTTTTAAATAGGAGAAGCCTTTATTTCAACCCCT
35[72]	33[71]	ACAGTTGTTGCTCCTTTTGATACATTGAAT
35[88]	3[87]	TTCTGCGAGCCTCAGAGCATAAAGGATTCAAAGTTAGAACCCTAAA

36[190]	37[190]	CCCTAGGTGGCATCACTATTTTTGAGCCCT
37[136]	35[135]	CAACCGTTATAAATCATACAGGCATTTCGCAA
37[7]	38[7]	CCCTGCAAGGATAAAAATTTTTAGTTAAATTGTAAACGTTAATACCCT
37[72]	35[71]	AGGTAAACTAAATCGGTTGTACTCCATATA
38[167]	6[168]	AGGTCATTTGCCGGAGAGGGTAGATTCTACATCCAGAAAAACGCTC
38[190]	39[190]	CCCTAGATCTACAAAGCGGATTGACCCCCT
38[39]	6[40]	GCAAATATAACCCTCATATATTTTCTGTAATAAGTGAGGCTCTGACCT
39[104]	37[103]	TGTAGCCAAAACTAGCATGTCAATAAAGGCCG
39[120]	0[104]	CAACATTTTGAGGGGACGACGATTTCCCAGCGAGCCGGAAGCATAA
39[136]	37[135]	GCGAGTAACAAACAAGAGAATCGAATGATATT
39[152]	0[136]	CGGATTCTGATGGGCGCATCGTAACAGTGCCATTGTTATCCGCTCACA
39[56]	0[40]	TTTAACCAGCTTCTGGTGCCGGAATATTACGCGCGTTGCGCTCACTGC
39[7]	40[7]	CCCTTTTTGTTAAAATTCGCATTATTCGCCATTCAGGCTGCGCACCCT
39[72]	37[71]	GCCATCAGATAATCAGAAAAGCTAATGTGT
39[88]	0[72]	TCGCGTCTAAGATCGCACTCCAGCTGCAAGGCTGCCTAATGAGTGAG
4[135]	6[136]	GCGCGTACTGAGTAGAAGAACTTCAATCGT
4[167]	34[168]	CACACCCGCTGGTAATTAATAGTAATTTTCATTTGGGGCCGAAAGA
4[195]	3[195]	CCCTGCGGTCACGCTGCGCGCGCTGGCAAGTGTACCCT
4[39]	34[40]	TAGACAGTATAATCCTTTTGCGTGCAACTAAAGTACGGGATGGCTT
4[71]	6[72]	GGAGCTAACTGTCCATCACGCAAAAGGGACAT
40[190]	41[200]	CCCTGTAATGGGATATCTAGAGGATCCCCGCCCT
41[104]	39[103]	GCCAGGGTCAGTATCGGCCTCAGGGGCCTTCC
41[136]	39[135]	ACGACGGCCCGTGCATCTGCCAGTAAATGTGA
41[168]	2[168]	GGTCGACGGTCACGCCACGCTGATGGTGGT
41[40]	2[40]	CTCTTCGCACCAGGCACGGCCAACGAAAAACC
41[7]	41[39]	CCCTACTGTTGGGAAGGGCGATCGGTGCGGGC
41[72]	39[71]	GATGTGCCAGCTTTCCGGCACCATAGGAAC
5[12]	4[12]	CCCTTCCTGAGAAGTGTTTTGAACGGTACGCCAGAACCCT
5[120]	39[119]	TCACTTGCCTATGGTTCATCAATTGAACGGTAATCGTAGCTTTCAT
5[152]	39[151]	CGGCCTTGCCGCGCTTTAAATTAAGCCTGAGAGTCTGGAGCAACCCGT
5[56]	39[55]	AAAAGAGTACAGGAGGTGCCTGAGCCCAAAAACAGGAAGAGCTCATTT
5[88]	39[87]	TTGTAGCACTTTCCTCAGGGTGAGCATATGTACCCCGGTTAAAATAAT
6[103]	8[104]	CCAGTCACCGAACCACCAGCAGAAAGGAATTG
6[135]	8[136]	CTGAAATGGCGGTCAGTATTAATATCTGGT
6[167]	8[168]	ATGGAAATGCCACGCTGAGAGCCATGCTGAAC
6[195]	5[195]	CCCTCCATTGCAACAGGAACAATATTACCGCCAGCCCT
6[39]	8[40]	GAAAGCGTGGCTATTAGTCTTTTCAATAGA
6[71]	8[72]	TCTGGCCAGCCCTAAAACATCGCCTAGGAGCA
7[12]	6[12]	CCCTAGACAATATTTTTGAATAAGAATACGTGGCACCCCT
7[120]	5[119]	AGAGGTGAGGATTATTTACATTGGTAATAACA
7[152]	5[151]	GCAACAGTACCTACATTTTGACGCCAAACTAT
7[56]	5[55]	AACTGATAACAGAGATAGAACCCTCACCGAGT
7[88]	5[87]	ATACCGAAACGACCAGTAATAAATTAACCG
8[135]	10[136]	CAGTTGGGAAGGAGCGGAATTACCATATCA
8[167]	30[168]	CTCAAATATATCAGATATACCACACAACATTATTACAGGACCAACT
8[195]	7[195]	CCCTTCTAAAGCATCACCTGCAGCAAATGAAAAACCCT

8[39]	30[40]	TAATACACAATTCGAAAGAAGTGCTTGAGATGGTTTAACATTCAGT
8[71]	10[72]	CTAACAACCGAACGTTATTAATTTACGTCAGA
9[12]	8[12]	CCCTTATTAGACTTTACAAATTTGAGGATTTAGAAGCCCT
9[120]	35[119]	AACCACCACAAATCAATCAAAAAAGACCGGAAGCAAACTGACCATT
9[152]	35[151]	TTCCTGATTCAAACCCAGTCAGAAATCGCGTTTTAATTCGTAACCTGT
9[56]	35[55]	CCTTTGCCTAATAGATTAAATATTAGAGGTCATTTTTGCGTGTCTGGA
9[88]	35[87]	TTGAGTAATATCTAAATAAACAGTTAGAGAGTACCTTTAAATTCCCAA

Modified oligonucleotides

Start	End	Sequence	Modification
29[104]	27[103]	TCGCCTGACGGCTACAGAGGCTTTTCTTAAAC/3ThioMC3-D/	Binding strands
33[136]	31[135]	TTACCCTGACATAACGCCAAAAGGAAAATCTA/3ThioMC3-D/	Binding strands
33[72]	31[71]	CCCCCTCAGACGACGATAAAAATGTGAATT/3ThioMC3-D/	Binding strands
37[104]	35[103]	GAGACAGTAAAATTAAGCAATAAAACGAGTAG/3ThioMC3-D/	Binding strands
11[120]	48[110]	TTAATTACTTGCACGTAAAACAGAAACAAAGAAAAAAAAAAA	Capture strands
11[88]	48[78]	AAGATGATAGATTTTCAGGTTTATAAAAGTAAAAAAAAAA	Capture strands
12[103]	53[113]	ACGCTGAGATTTCATCTTCTGACCTTGAGAATAAAAAAAAAA	Capture strands
15[120]	52[110]	TCCCATCCCAACAGTAGGGCTTAATAAATTTAAAAAAAAAAA	Capture strands
15[88]	52[78]	CAATAGATTTAACAACGCCAACAAATATATAAAAAAAAAA	Capture strands
27[120]	50[110]	TTGCGCCGAACGAGGGTAGCAATAAATTGTGATAGCTTAAAC AAAAA	Capture strands
27[88]	50[78]	GGTGAATTGAGGACTAAAGACTTTAAAGTACAATAGTGAGCA AAAGAAAAAAAAAA	Capture strands
31[120]	46[110]	GGGAAGAAATTACGAGGCATAGACCATAAACAGTTGAAGATA AAACAAAAAAAAAA	Capture strands
31[88]	46[78]	AAGAACTGATAACCCTCGTTTACCAAATGCTTATATCTTATT AAAAAAAAAAAAAA	Capture strands
4[103]	45[113]	ATAACGTGATACTTCTTTGATTAGCAGATTCAAAAAAAAAAA	Capture strands
10[103]	12[104]	AATTGCGTGAAACAAA/iCy5/CATCAAGAAGATTAAG	Internal Cy5
8[103]	10[104]	AGGAAGGTCATTATCA/iCy5/TTTTGCGGAATAAAGA	Internal Cy5
		/5ThioMC6-D/TTTTTTTTTTTTTT	AuNP functionalisation

S3. Fluorescence emission from dye-labelled DNAo

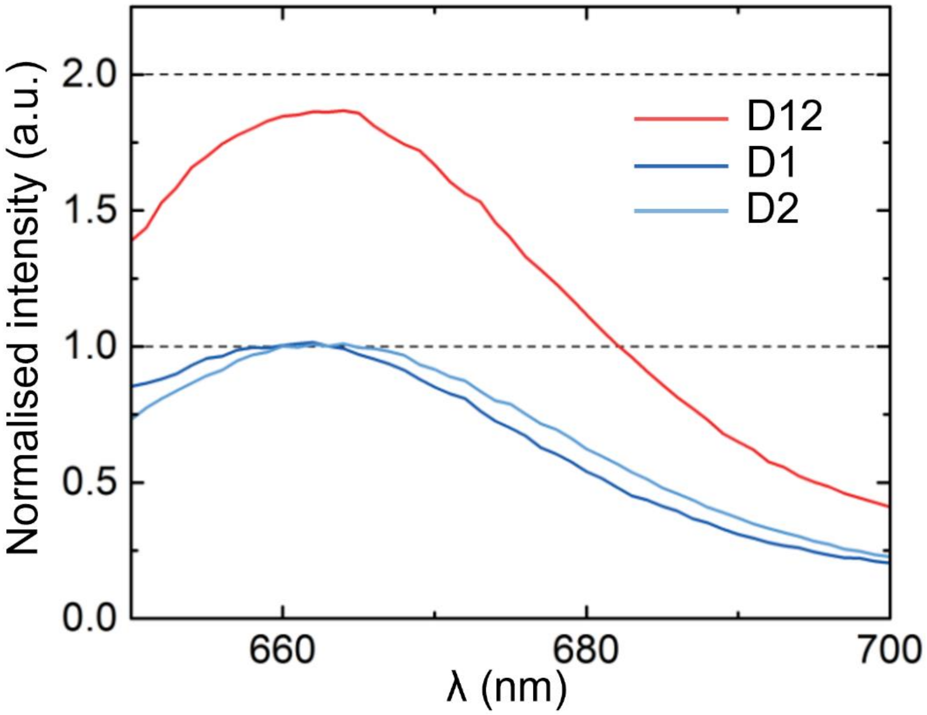

Figure S3. Bulk fluorescence emission from dye-labelled DNAo. Fluorescence intensity from DNA nanostructures containing a single quantum emitter (D1 or D2, blue and light blue curves) is significantly lower than fluorescence intensity from DNA nanostructures containing simultaneously 2 quantum emitters (D12, red curve).

S4. AFM images with different scale bars

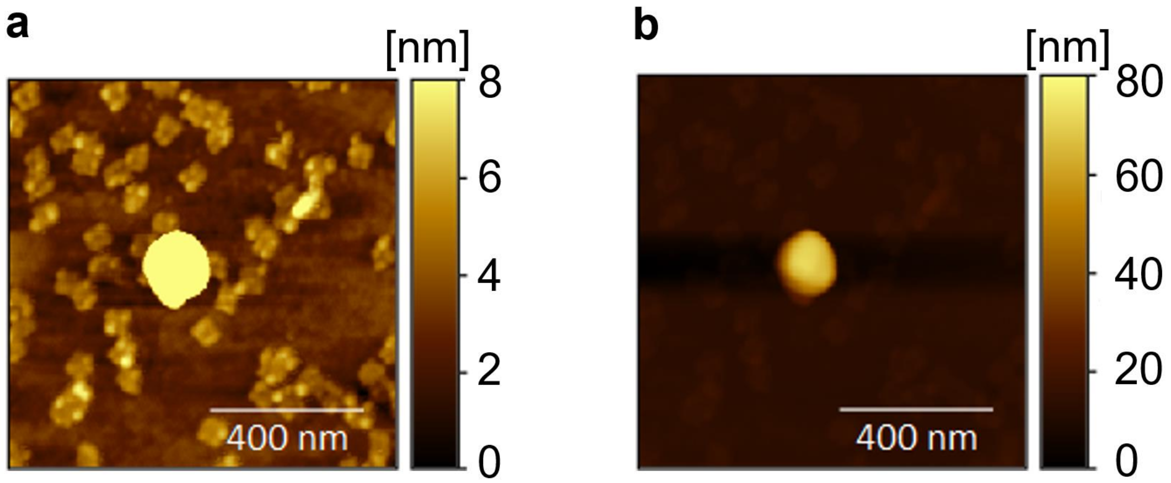

Figure S4. AFM images at different scale bars. The same AFM image is represented with two different scale bars (0-8 nm in **a** and 0-80 nm in **b**) to better resolve the size of the AuNP bound onto DNAo. Because of the significant size difference between the DNAo and the AuNP, the former are not visible in **b**.

S5. Dark-field measurements to control AuNP immobilisation on DNAo

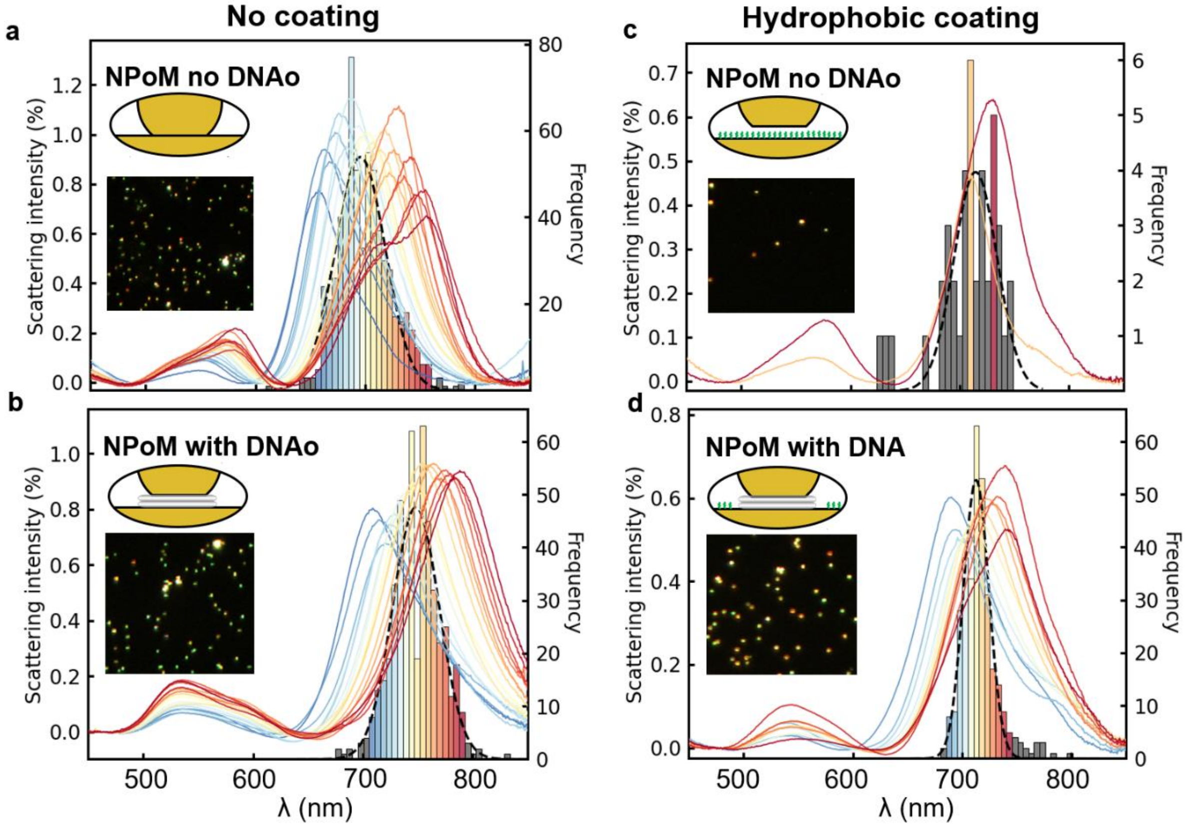

Figure S5. Dark-field histograms of different NPoM conditions. AuNP were drop cast on a bare (**a** and **b**) or dodecanethiol coated (**c** and **d**) Au surface. DF images (black insets) show significantly higher binding efficiency of AuNP on bare Au than on dodecanethiol coated Au. DF histograms confirm such evidence: in **a** and **b** the distribution of resonance modes spreads for over 90 nm, suggesting that AuNP are binding to both bare Au and DNA nanostructures with no preference. On the contrary, in **c**, the dodecanethiol coat prevents AuNP from attaching to gold (non-homogeneous and wide histogram distribution). In **d**, the DF histogram distribution becomes considerably narrower than **a**, **b** and **c**, indicating that AuNP are only binding to DNA nanostructures, rather than on the bare dodecanethiol-coated Au.

S6. DLS measurements of DNA-functionalised AuNP

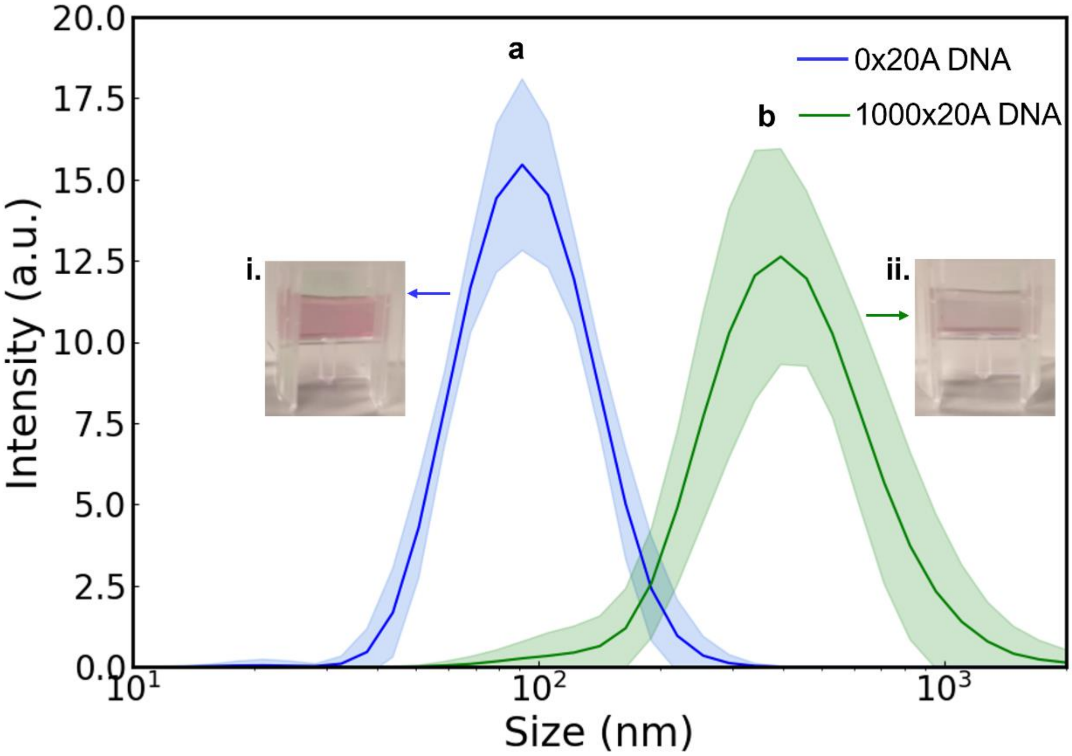

Figure S6. DLS measurements of DNA coated AuNP. Commercial AuNP (*D* = 80 nm) were coated with an excess of thymine nucleotides (14T-SH) and purified according to the protocol described in Methods and Materials (S13). The concentration of $MgCl_2$ was adjusted to 10 mM (**a**). The size distribution is in the expected range and the pink colour accounts only for the dilution factor. A 1000x excess of single-stranded DNA (20A) was added to verify if the thymine strands were available for binding. After 2 minutes, the colour changed to grey (ii.) and the size distribution increases drastically, suggesting that the complementary DNA strands are able to bridge the colloids together. This result confirms that the protocol to functionalize AuNP with DNA lead to a good coverage of DNA strands around the colloids, and further confirms that the DNA strands are available to bind on to the capture strands of the DNAo.

S7. Fluorescence quenching by AuNP

To verify the binding of AuNP to DNA nanostructures, we studied the quenching of fluorescence of quantum emitters induced by metal colloids.[1] Bulk solution fluorescence from dye-labelled DNAo after λ_p = 633 nm irradiation was recorded. DNA-coated AuNP (*D* = 10 nm) were then added to the same cuvette and the fluorescence over a time of 120 min was measured. DNAo nanostructures were folded with (red curve) and without (grey curve) capture strands. In presence of capture strands with polyA overhangs, the fluorescence decreases over time, suggesting the binding of AuNP to DNAo in proximity of the dye molecules. In absence of capture strands with polyA overhangs, the bulk fluorescence remains constant. To avoid quenching of fluorescence induced by proximity of AuNP to dyes, the excess of AuNP to DNAo was planned so that statistically a single nanoparticle only would be able to bind to each nanostructure.

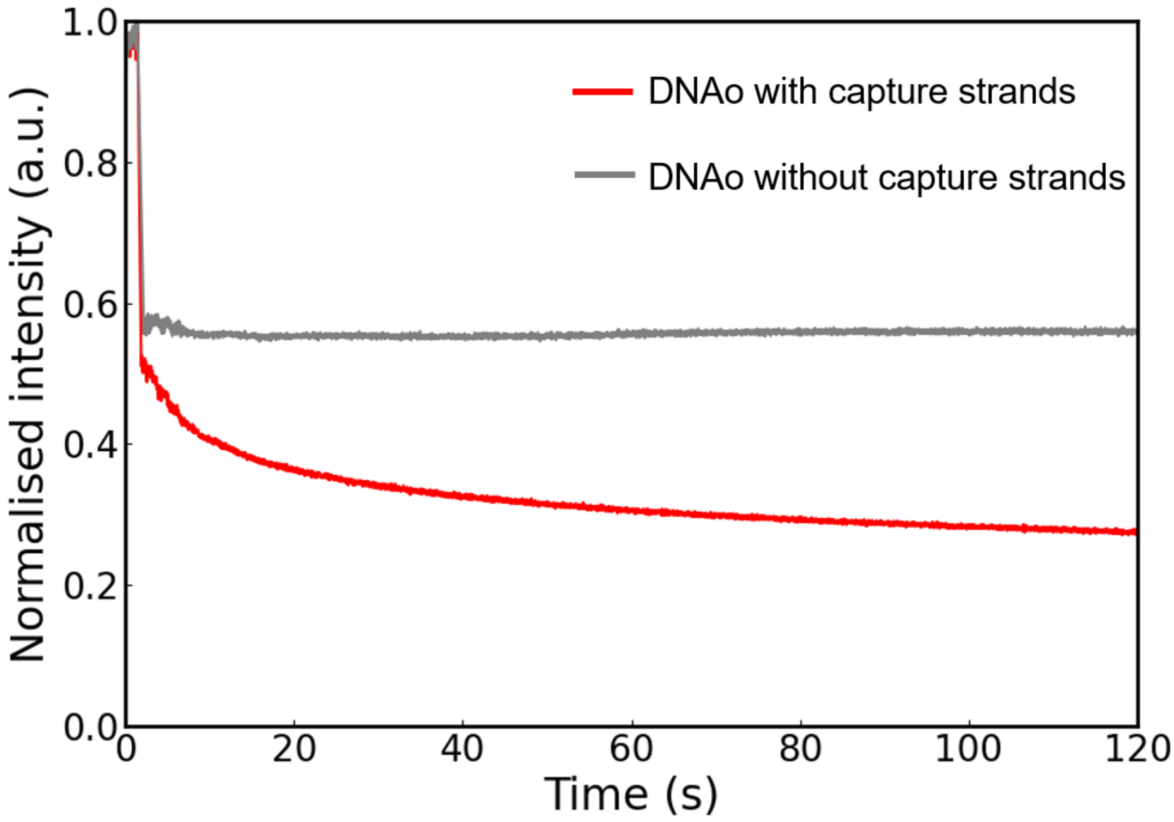

Figure S7. Fluorescence quenching. Bulk solution fluorescence from dye-labelled DNAo at λ_p = 633 nm was recorded. DNA-coated AuNP (*D* = 10 nm) were added to test for the quenching of fluorescence induced by the binding of AuNP to DNAo. DNAo nanostructures were folded with (red curve) and without (grey curve) capture strands to bind to AuNP. A time-dependent fluorescence intensity decrease is only observed when capture strands are present on DNA nanostructures.

S8. Light emission at lower power

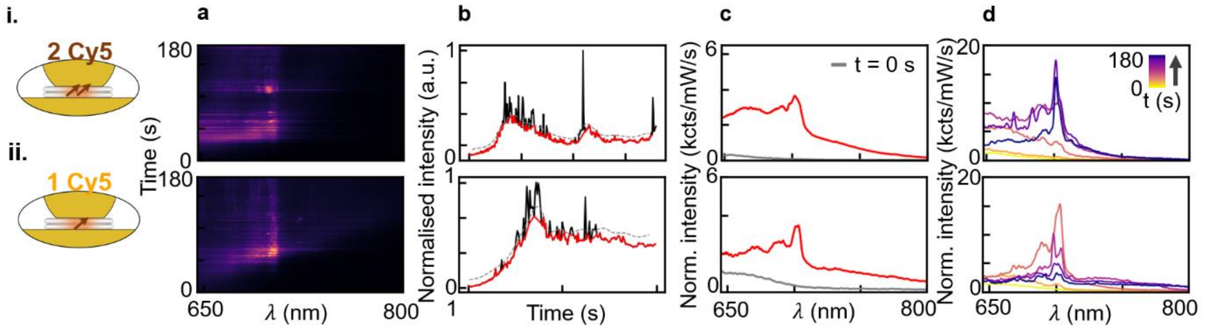

Figure S8. Light emission at low power. NPoM with 2x and 1x Cy5 molecules on DNAo were measured at 0.2 mW/µm^2, confirming the same behaviour as described in the main section of the manuscript. Time scans (**a**) and spectrally-integrated emission intensity *vs* time (**b**) show gradual increase of light emitted from dye-labelled NPoM. **c** Average emission from spectra without intensity spikes (red, **c**) and at *t*=0 (grey, **c**) and **d** emission spectra *vs* wavelengths every 30 s (colour denotes time).

S9. ATTO647N

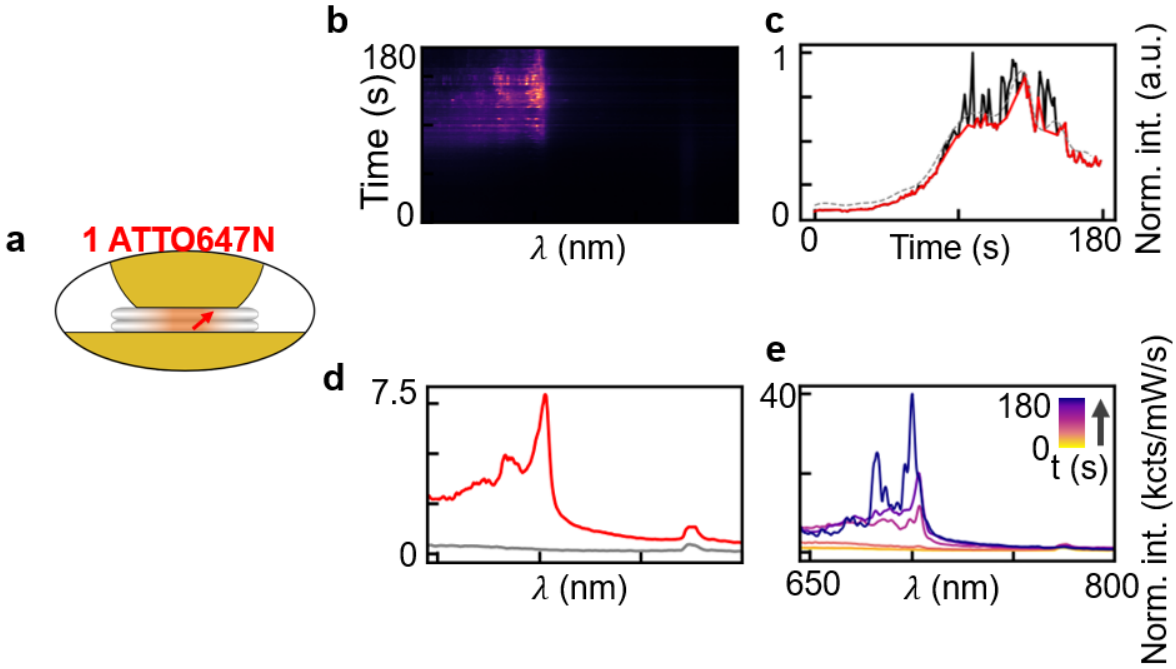

Figure S9. ATTO647N. The Cy5 molecules on the DNA nanostructures were replaced with the ATTO647N dye. Despite having a different molecular structure, the NPoM emission spectrum does show similar features as the cyanine dyes. **a** NPoM with ATTO647N instead of Cy5 as a quantum emitter on DNAo, **b** SERS time scan and integrated emission intensity over time (**c**). Extracted averaged emission spectrum from red in **c** (**d**) and at t=0 (grey in **c**) and emission spectra *vs* wavelength every 30 s (colour denotes time).

S10. Long-lived states

In order to investigate the role of light in the appearance of spectral features, we studied the DNAo NPoM at different intervals of light illumination. Samples were irradiated at λ_p = 633 nm for 30 s, after which both white light and laser light were turned off for different time intervals (1 minute in ii., iii. and iv., 2 minutes in v. and 5 minutes in vi.). At initial times, there is light-induced slow increase in emission (i. and ii.), that does not return to zero when light is off. After the appearance of the typical spikes (iii. and onwards), these remain present even when light is turned off, suggesting the presence of long-lived states.

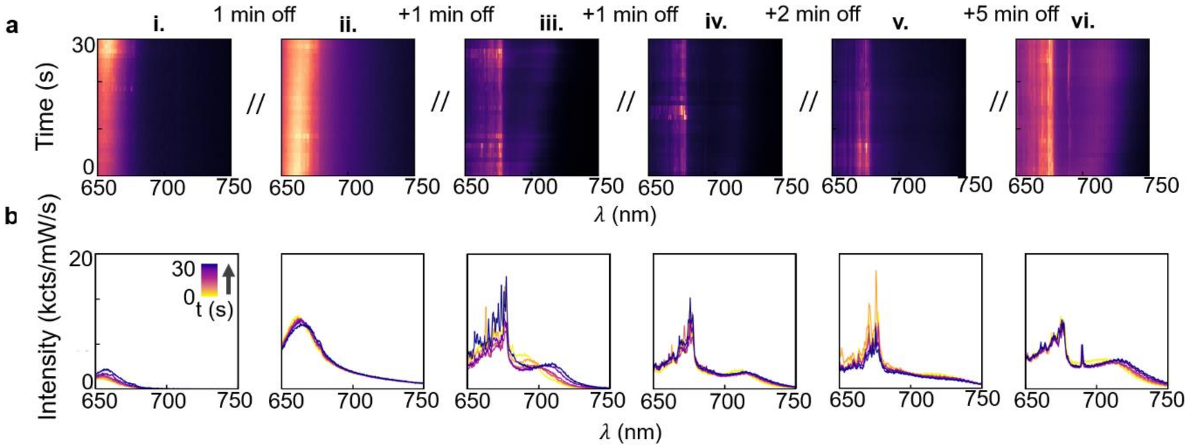

Figure S10. Long-lived states. Time-dependent SERS maps (**a**) of NPoM with dye-labelled DNAo as a spacer and their spectral behaviour in wavelength (**b**). Samples are irradiated at 0.7 mW/µm^2 for 30 s each time, after which the laser is turned off for the time indicated on the top panel. Making sure to be centre at the same particle, light is then turned on again for further 30 s (colour denotes time).

S11. Wet NPoM

So as to exclude any light-induced drying effect of the NPoM, those were investigated in a wet environment. Samples were immobilised on Au as described in Methods and S13 c) and measured using a home-built flow-cell. Such measurement allows the observation of the dye-NPoM system in a buffer solution. As for the dry state, the results confirm the time time-dependent appearance of SERS lines, which indeed confirms that the DNA-bound water molecules stay confined in the plasmonic cavity, even after long irradiation times.

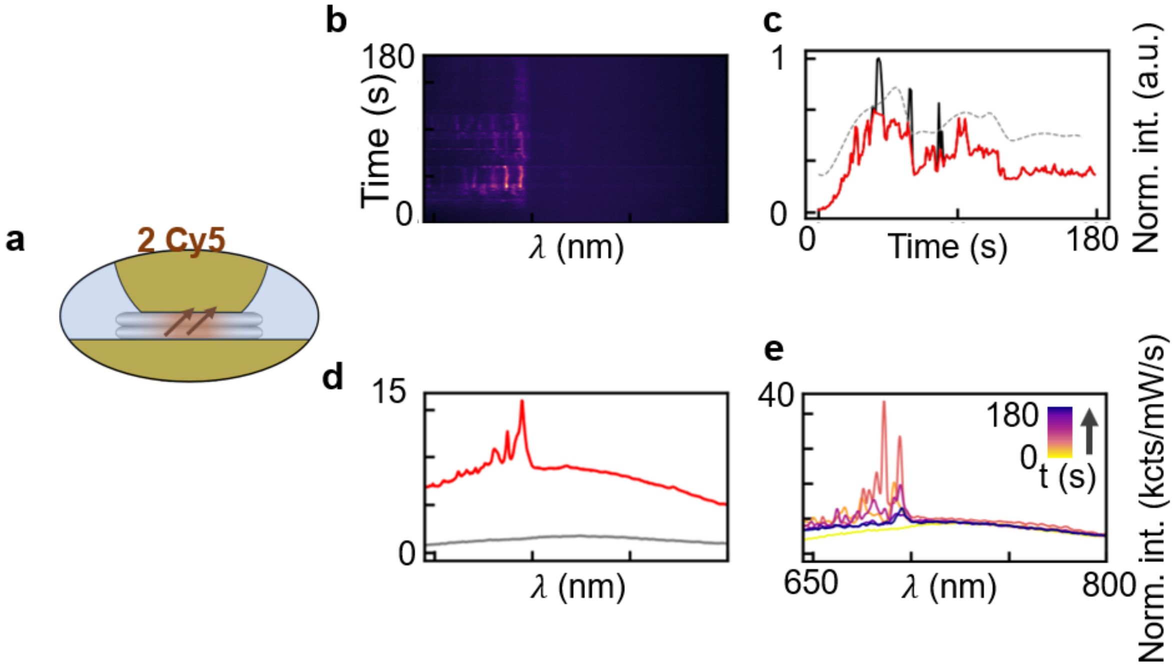

Figure S11. Wet NPoM. DNAo with 2x Cy5 (**a**) was used as a NPoM spacer and immobilised in a home-built flow cell. Measurements were carried out in 1xTE, 12 mM $MgCl_2$. Time-dependent SERS maps (**b**) and integrated over time (**c**) and time-series spectra *vs* wavelength (**d**) show similar behaviour to the dry sample.

S12. Dark-field before and after laser illumination

DF spectra before and after laser illumination were recorded in order to confirm that no effects coming from shrinking of the gap size occur. Here we report DF spectra before and after 633 nm pumping coming from three different NPoMs. In all cases, both scattering intensity and peak position remain unchanged, confirming that no shrinking of the plasmonic gap is observed and the integrity of the DNA plate.

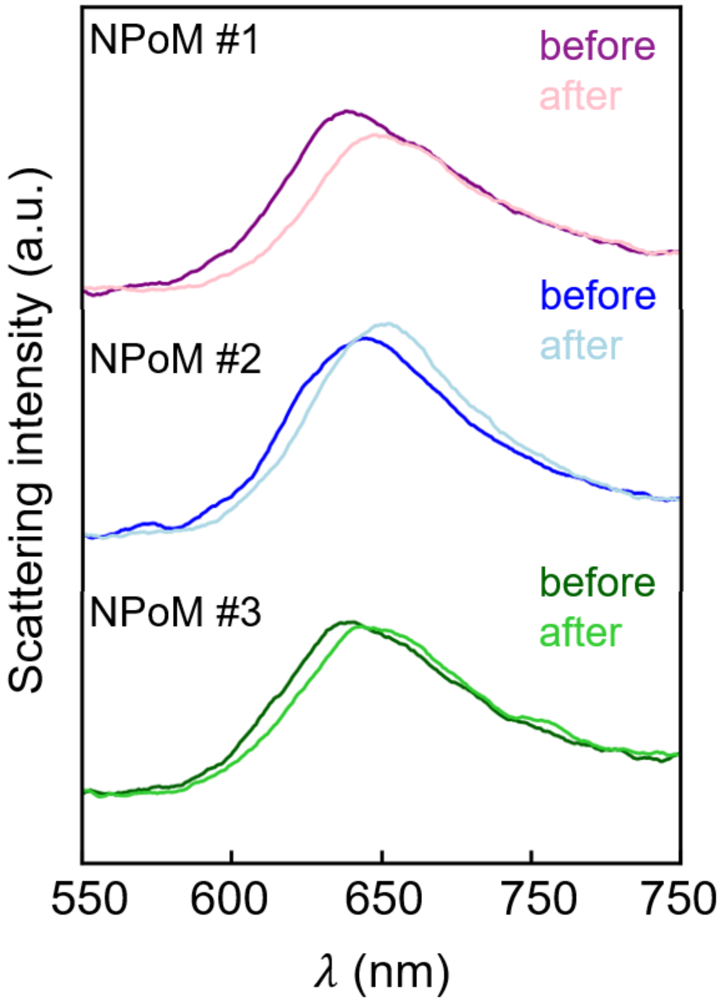

Figure S12. DF spectra before and after laser illumination. In order to check for the integrity of the NPoM cavity and gap size after laser illumination, spectra from different NPoMs (*D* = 60 nm) are reported. In all three cases, the DF scattering spectrum remains unchanged.

S13. Methods and materials

a) Details of DNAo design Each layer of the double plate contains 21 helices. The DNA nanostructure is folded from one circular single-stranded DNA scaffold and 186 customised staple strands of average length of 37 nucleotides. On the top layer, ten capture strands (average length of 47 nucleotides) are designed to complementary bind to AuNP. Each of these ends with 10 adenine bases on the 3' side. On the bottom layer, binding strands (average length of 31 nucleotides) serve to immobilise the nanostructure on the Au. Each of these ends with a dithiol functionalization on the 3' side.

b) DNAo assembly Single-stranded M13mp18 viral DNA (7249 nucleotides) was purchased from Guild BioSciences. All unmodified and modified staples were acquired from IDT DNA. The assembly mix contained a final concentration of 10 nM scaffold DNA with a 10-fold excess of unmodified and a 15-fold excess of modified staple strands (i.e. dye- or thiol-labelled strands) and was carried out in a buffer containing 1xTE, 12 mM $MgCl_2$. An annealing cycle from 70 °C to 4 °C is carried out over the course of 16 h on a ProFlex PCR thermocycler, followed by a holding period at 4 °C.[2] Subsequently, assembled DNAo plates were purified from excess staples using centrifugal filters (Amicon, cutoff 100 kDa).

b) Gold nanoparticles functionalization Single-stranded DNA (IDT DNA) is used to functionalize AuNPs. Each strand consists of 14 thymine nucleotides and ends with a dithiol group on the 5' side. For PL and SERS measurements, superspherical citrate-capped, gold nanoparticles (D = 60 nm) were synthesized according to [3]. For DF and AFM imaging of NPoMs, D = 80nm citrate-capped gold nanoparticles were purchased from BBI. In both cases, a 0.1 mM aqueous solution of AuNP is mixed with 100 µM 5'-thiol modified 14× poly thymine DNA strands (65000x excess DNA on AuNP). To ensure binding of the single stranded DNA molecules onto the nanoparticle surface, magnesium chloride is used as a salt-ageing agent. The salt concentration is gradually adjusted from 0 to 8 mM in 40 steps in 80 min. Then, DNA-functionalized gold nanoparticles are purified via centrifugal sedimentation and kept in 1xTE buffer. For DNAo immobilisation, DNA-functionalised AuNP are diluted in 1xTE, 12 mM $MgCl_2$.

c) NPoM assembly DNA nanostructures (40 µL, 2 nM) are drop cast on a template-stripped gold film and incubated for at least ten minutes. After this, the metal film is rinsed with MilliQ water and immersed in a 1 mM solution of dodecanethiol (Sigma-Aldrich) in ethanol overnight. After rinsing the substrate with MilliQ water and drying it with a stream of nitrogen, DNA-functionalized AuNP (20 µL, 0.01 nM in 1xTE, 12 mM $MgCl_2$) are drop cast on the metal surface and incubated for at least five minutes. Then, the substrate is rinsed with MilliQ water and dried with a stream of nitrogen.

d) Solution fluorescence measurements Dye-labelled DNA structures (60 uL, 2 nM in 12mM $MgCl_2$, 1xTE buffer) are imaged on a Fluorimeter (Cary300Bio) using an excitation wavelength of 542 nm for Cy3 and 633 for Cy5.

e) Single nanoparticle DF and SERS measurements Both DF and SERS spectra are recorded on a home-built confocal Raman microscope. The signal is split into two channels and read out with an Ocean Optics spectrometer (for DF) and Andor EMCCD (for SERS). To select the optical signal from a single nanoparticle, the slit width and readout lines on the EMCCD are limited. A Lumenera Infinity2 camera is used to acquire DF images. A Labsphere Spectralon reflectance standard is used to correct for efficiency of both collection and light sources in DF spectra. An Olympus DF objective of NA=0.9 is used to ensure light collection at high angles. The 633 nm single-frequency He-Ne laser is coupled into

the microscope and aligned to focus on the same area of the sample during each single-molecule time scan. The laser power is measured using a Thorlabs PM16-121 power meter after passing through the microscope objective. The Raman signal from undoped silicon wafers (521 cm^{-1}) is used to calibrate the SERS intensity and wavelength in all spectra. An in-house algorithm implemented in python is used to carry out a particle tracking that simultaneously controls all the computer-controlled instruments i.e. motorized sample stage, spectrometer and laser. To record DF spectra, an automatic scan through the focal depth of each nanoparticle is performed with integration times of 1 s. The scattering spectrum corrected from chromatic aberration is extracted using a Gaussian fitting of each depth-dependent intensity at each wavelength.

References

(1) Kongsuwan, N.; Demetriadou, A.; Chikkaraddy, R.; Benz, F.; Turek, V. A.; Keyser, U. F.; Baumberg, J. J.; Hess, O. Suppressed quenching and strong-coupling of purcell-enhanced single-molecule emission in plasmonic nanocavities. *Acs Photonics* **2018**, *5* (1), 186-191.
(2) Kopperger, E.; List, J.; Madhira, S.; Rothfischer, F.; Lamb, D. C.; Simmel, F. C. A self-assembled nanoscale robotic arm controlled by electric fields. *Science* **2018**, *359* (6373), 296-301.
(3) Lee, Y.-J.; Schade, N. B.; Sun, L.; Fan, J. A.; Bae, D. R.; Mariscal, M. M.; Lee, G.; Capasso, F.; Sacanna, S.; Manoharan, V. N. Ultrasmooth, highly spherical monocrystalline gold particles for precision plasmonics. *ACS Nano* **2013**, *7* (12), 11064-11070.
